# Assessment of association of exposure to polycyclic aromatic hydrocarbons with bronchial asthma and oxidative stress in children: A case control study

**DOI:** 10.4103/0019-5278.50722

**Published:** 2009-04

**Authors:** Ram Suresh, Awasthi Shally, A. A. Mahdi, D. K. Patel, V. K. Singh, Misra Rita

**Affiliations:** Department of Pediatrics, Chhatrapati Shahuji Maharaj Medical University, Lucknow - 226 003, UP, India; 1 Department of Biochemistry, Chhatrapati Shahuji Maharaj Medical University, Lucknow - 226 003, UP, India; 2 Department of Analytical Toxicology Section, Industrial Toxicology Research Centre, P.O. Box 80, M.G. Marg, Lucknow - 226 001, UP, India

**Keywords:** Asthma, children, India, phenanthrene, polycyclic aromatic hydrocarbons, oxidative stress

## Abstract

**Background:**

Polycyclic aromatic hydrocarbons (PAH) originate from the incomplete combustion of organic matter and ambient air pollution by these is increasing. There is also an increase in the global prevalence of asthma, for which environmental pollution has been recognized as one of the important factors. Exposure to pollutants and other allergens induces chronic airway inflammation by generation of reactive oxygen species, causing oxidative stress. Therefore, the objective of the present study was to assess association, if any, between exposure to PAH and asthma as well as oxidative stress in children.

**Method:**

In this hospital-based case control study, cases of bronchial asthma aged 1–14 years and healthy matched controls were included. Oxidative stress was measured by assessing the levels of enzymes catalase, superoxide dismutase, malondialdehyde (MDA), and reduced glutathione (GSH).

**Results:**

Forty-two cases and 20 controls were enrolled. Mean blood level of phenanthrene, a PAH, was 63.11 ppb ± 115.62 and 4.20 ppb ± 10.68 ppb in cases and controls, respectively (*P* = 0.02). Mean blood levels of GSH was significantly lower in cases and controls (27.39 μg/ml ± 11.09 versus 47.39 g/ml ± 13.83; *P*-value = 0.001). Likewise, mean blood level of MDA in nanomole/ml was significantly higher in asthma as compared with controls (12.85 ± 5.40 versus 8.19 ± 5.16; *P*-value = 0.002), suggestive of increased oxidative stress.

**Conclusions:**

Because elevated blood level of phenanthrene is associated with bronchial asthma as well as with oxidative stress, measures to reduce exposure to PAH may possibly lead to reduced incidence and severity of bronchial asthma.

## INTRODUCTION

Asthma is the most common inflammatory lung disease in children and is a major public health problem, showing steady increases in prevalence both in developing and in developed countries.[1] Globally, 150 million people are suffering from asthma.[2] In India, there is wide variation (4–19%) in the prevalence of asthma in school going children from different geographic areas.[3] About half to one-third of the cases of asthma have been associated with ambient air pollution.[4]

Exposure to polycyclic aromatic hydrocarbons (PAH) as a result of ambient air pollution has also been associated with asthma. PAH are a group of small organic compounds of three to five benzene rings that are formed during the incomplete combustion of coal, oil, gas wood garbage, or other substances, such as tobacco.[5] Our hypothesis was that exposure to PAH results in an oxidative stress in the respiratory tract, which may stimulate the redox cycle and generate reactive oxygen species, resulting in exacerbation of airway inflammation and manifesting as asthma.[6]

The objective of the current study was to assess the association, if any, between exposure to PAH and bronchial asthma along with evidence of oxidative stress, as assessed by levels of antioxidant enzymes catalase (CAT), superoxide dismutase (SOD), glutathione (GSH), and malondialdehyde (MDA).[7]

## MATERIALS AND METHODS

### Study design

This was a hospital-based case control study carried out after obtaining institutional ethical clearance in a tertiary care hospital in Lucknow, northern India, from August 2005 to July 2006.

### Subjects

Inclusion criteria for cases were clinical diagnosis of bronchial asthma, defined as a history of more than three episodes of documented wheeze in a year.[8] Inclusion criteria for controls was no history of asthma or any concomitant respiratory problem and were age, sex, weight for height (World Health Organization Z score), and residential area matched healthy children. Excluded were cases and controls with congenital heart disease, chronic lung disease like suspected cystic fibrosis, bronchiectasis, lung abscess, or whose parents or guardian did not consent to participation.

### Sample size calculation

To assess exposure of 50% and 10% of cases and controls, respectively, to any PAH with an alpha level of 0.05 and power of 0.8 with a 2:1 ratio of cases to controls, 18 controls and 37 cases were to be recruited.

### Variables for data collection and definition

Data were collected on age, sex, anthropometry, weight, height, family characteristics, residential area, immunization status, history of allergy, family history of asthma, and surrogate markers of exposure to environmental pollutants, like number of rooms in house, separate cooking space, use of biomass fuels for cooking, smoking status of parents, distance of residence from heavy traffic, and industrial factory in near by area. Exposure to PAH and measurement of oxidative stress were assessed by biochemical analyses of venous blood.

### Biochemical analysis

Approximately 5.0 ml of venous blood was withdrawn from all subjects and was stored in pre-heparinized glass vials. Samples were coded and transported under ice-cold conditions to the Analytical Toxicology Lab, Industrial Toxicology Research Centre, Lucknow, for analysis of PAH and antioxidant enzymes. The analytical toxicologist was totally blind to the medical history and final diagnosis of the subjects. Two milliliters of blood was used for the preparation of the lysate. Blood was centrifuged at 2500 g for 15 min at 4°C and the supernatant was aspirated. The erythrocyte-rich precipitate was washed three times with ice-cold physiological saline (3:1 v/v) and lysed by double distilled water. The particulate material was centrifuged at 15,000 g for 90 min at 4°C and the supernatant (erythrocyte lysine) was collected for determination of CAT and SOD activity. Three milliliters of blood was utilized for the estimation of MDA, GSH, and PAHs levels.

#### Analyses of PAH

Extraction of PAH from blood was carried out according to Van Scooten *et al*.[9] Peak confirmation of PAHs in blood samples was performed by gas chromatography (mass spectrometer using model auto system XL [Perkin-Elmer _Waltham, Massachusetts,_ USA] coupled with a Turbo Mass detector). The sample was reduced to 0.5 ml under a gentle stream of nitrogen and analyzed on an HPLC–FD at a reverse phased (RP) C - 18 ODS analytical column (75 mm × 4.6 mm i.d., 3.5 μm particle size), with a pre-column of the same phase from Waters (Water Milford, MA, USA) for different PAHs other than acenaphthylene (due to no response by FD). The elution conditions and detection wavelength program was same as that reported by Barranco *et al*.[10] Recovery experiments were conducted to check the analytical quality control. Samples of blood were spiked with a mix standard of 16 PAHs and the recoveries ranged from 78 to 94% for all PAH compounds. Gas Chromatography conditions and temperature programming was same as described by Poon *et al*.[11] The mass detector was operated in an electron impact at 70 eV in full scan. The target compounds were identified in the scanning mode. The spectrum of individual PAH was confirmed by matching it with the authentic spectra of standard PAH in the list library available in the GC-MS instrument Perkin-Elmer _Waltham, Massachusetts,_ USA.

According to the Environmental Protection Agency priority list, the following PAH were studied: naphthalene, acenapthene, phenanthrene, anthracene, fluoranthene, pyrene, benzo (b) fluo, benzo (k) fluo, benzo (a) pyrene, di benzo (a,h) anthracene.

#### Estimation of antioxidant enzymes

The extent of lipid peroxidation was estimated by measuring the formation of MDA by the reaction of MDA with thiobarbituric acid and expressed as nmol MDA/ml blood, using a molar extinction coefficient of 1.56×l0^5^ M^−1^ cm^−1^.[12] Reduced GSH was estimated in the whole blood using Ellman's reagent and expressed as *μ*mol/ml of blood.[13] CAT activity was determined using hydrogen peroxide (H_2_O_2_) as substrate and expressed as *μ*mol H_2_O_2_ decomposed/min/g hemoglobin (Hb).[14] SOD activity determination was carried out by the method of Mishra *et al*.[15]

### Data management

Data were entered in MS-Excel 2000. Statistical analysis was performed using the Statistical Package for Social Sciences for windows (SPSS-Version 11, Chicago, Illinois, USA). Univariate analysis was performed to study the frequency distribution of the variables. χ^2^ and Students *t*-test were used to test the association between categorical and continuous variables, respectively. The Kruskal Wallis test, a non parametric test equivalent to one way ANOVA (F test), was used to check whether the mean level of PAH differs significantly among cases and controls. In order to asses the effects of PAH exposure by different sources and other potential risk factors for asthma, the crude odds ratio (OR) with 95% confidence interval (CI) was calculated. For those variables that had a univariate association with asthma, multivariate logistic regression analysis was performed to calculate the adjusted OR with 95% CI. Statistical inferences were based on the conventional level of statistical significance (*P* < 0.05).

## RESULTS

Sixty-two children, 42 cases of bronchial asthma and 20 controls, were included. Demographic features are given in Table 1. The male to female ratio was 1.9:1. Family characteristics and environmental exposures among cases and control are given in Table 2. Non-availability of separate cooking space, father smoking status, and history of allergy were positively associated with asthma. Family type, use of biomass fuels, and residential area distribution was not significantly associated with case control status.

**Table 1:** Demographic features among cases and controls

	Case n. % (*n* = 42)	Control n. % (*n* = 20)	χ^2^, *P*-value
Age (in years)			
1–5	29 (69.04)	14 (70.0)	0.226, 0893
5–10	10 (23.80)	4 (20.0)	
10–14	3 (7.16)	2 (10.0)	
Sex			
Male	27 (64.29)	14 (70.0)	0.198 0.657
Female	15 (35.71)	6 (30.0)	
WHO “Z” Score –	10 (23.80)	4 (20.0)	
weight for height			
-3 s.d to -2 s.d			
<-3 s.d.	2 (4.0)	1 (5.0)	

WHO “Z” Score – weight for height, adequately nourished: WHO “Z” -1. s.d., standard deviation. Malnourished: WHO “Z” score <-1.

**Table 2:** Family characteristics and environmental exposures among cases and controls

Characteristics	Cases (*n* = 42)		Control (*n* = 20)		OR, 95% CI	*P*-value
		
	No.	%	No.	%		
Urban residence	22	52.40	12	60.0	0.7 (0.2–2.2)	0.57
Unimmunized	10	23.80	4	20.0	1.2 (0.3–4.6)	0.74
Joint family	11	26.20	9	45.0	0.4 (0.1–01.5)	0.14
No separate cooking space	25	59.5	3	15.0	8.3(2.1–32.9)	0.001
Father's smoking	29	69.1	4	20.0	8.9 (2.2–39.7)	0.002
Biomass fuels other than LPG	18	42.9	7	35.0	1.4 (0.5–4.2)	0.55
History of allergy	21	50.0	3	15.0	5.7 (1.3–28.7)	0.002

LPG, liquefied petroleum gas. Biomass fuels: coal, wood, dung cake, kerosene.

Proportion of cases and controls positive for PAH in blood samples as well as their mean levels are given in Table 3. Mean blood level of phenanthrene was statistically significantly higher in cases (63.11 ppb ± 115.62) as compared with controls (4.20 ppb ± 10.68; *P* = 0.02).

**Table 3:** Comparison of mean blood levels in ppb of polycyclic aromatic hydrocarbons of cases and controls

Polycyclic aromatic hydrocarbons	Cases (*n* = 42)		Control (*n* = 20)		*t*-test, *P*-value
		
	n, %	Mean ± SD (min.–max.)	n, %	Mean ± SD (min.–max.)	
Napthalene	21 (50.0)	69.90 ± 105.81 (0–432)	11 (55.0)	110.8 ± 244.22 (0–1099)	0.14, 0.35
Acenapthene	22 (52.4)	147.16 ± 246.112 (0–958)	6 (30.0)	326.1 ± 861.99 (0–3064)	1.25, 0.215
Phenanthrene	29 (69.0)	63.11 ± 115.621 (0–524.82)	20 (15.0)	4.20 ± 10.68 (0–34.15)	2.26, 0.027
Anthracene	24 (57.1)	3.002 ± 5.471 (2–26.53)	0 (0)		
Fluoranthene	20 (47.6)	16.90 ± 27.230 (0–89)	8 (40.8)	38.2 ± 82.47 (0–330)	152, 0.13
Pyrene	19 (45.2)	22.56 ± 49.643 (0–278.28)	5 (25.0)	41.58 ± 153.32 (0–689.86)	0.73, 0.47
Benzo(b)fluo.	7 (16.7)	2.08 ± 5.791 (0–26)	0 (0)		
Benzo(k)fluo.	4 (9.5)	26.75 ± 104.071 (0–483.20)	0 (0)		
Benzo(a) pyrene	4 (9.5)	0.32 ± 1.137 (0–5)	2 (10.0)	0.45 ± 1.40 (0–5)	0.42, 0.68
Di benz (a,h)	1 (2.4)	0.45 ± 2.920 (0–19)	0 (0)		

Ppb, parts per billion.

Comparison of mean blood levels of antioxidant enzymes, CAT, and SOD, as well as GSH and MDA between asthma and controls is given in Table 4. Mean levels of GSH in blood were significantly lower in cases as compared with controls. Mean level of MDA in nanomole/ml was significantly higher in asthma as compared with controls. Mean blood levels of CAT and SOD were similar among cases and controls.

**Table 4:** Comparison of antioxidant level in blood samples of cases and controls

Antioxidant enzymes	Cases (*n* = 42) Mean ± SD (min.–max.)	Control (*n* = 20) Mean ± SD (min.–max.)	*t*-test, *P*-value
GSH (μg/ml) of blood	27.39 ± 11.09 (12.14–53.70)	47.39 ± 13.83 (18.21–68.74)	6.12, 0.000
MDA/nmol TABRS ml blood	12.85 ± 5.40 (4.35–25.38)	8.19 ± 5.16 (2.42–16.38)	3.22, 0.002
Catalase (μ mol H_2_O_2_ hydrolyzed/min/g Hb)	929897.99 ± 339614.77	800612.16 ± 374358.54	
	(293763.53–1694221.1)	(57.99–1541871.0)	
SOD (units/min/mg Hb)	32412.97 ± 37731.72	30015.45 ± 43057.26	0.22, 0.82
	(4711.08–156568.6)	(5528.96–204962.6)	

Values in the parenthesis are minimum and maximum. GSH, reduced glutathione; SOD, superoxide dismutase; TBARS, thiobarbituric acid; MDA, malondialdehyde; H_2_O_2_, hydrogen peroxide.

Multivariate conditional logistic regression analysis was performed to assess the association of blood PAH and oxidative stress with case control status, controlling for other variables that either had a univariate association with it or were clinically meaningful or could be potentially modified. Variables associated with bronchial asthma were father smoking status (adjusted OR = 7.4, 95% Cl: 1.2–43.7; *P* = 0.03), mean blood levels of phenanthrene (adjusted OR = 13.3, 95% Cl: 1.9–88.5; *P* = 0.008), and GSH (adjusted OR = 0.8, 95% Cl: 0.8–0.9; *P* = 0.001).

## DISCUSSION

In the present study, we assessed the exposure to PAH by estimating their levels in blood and also evaluated their possible association with bronchial asthma as well as with oxidative stress. We found a higher blood level of phenanthrene as well as evidence of oxidative stress, in the form of reduced blood level of GSH and elevated blood MDA level, in cases of bronchial asthma when compared with matched controls.

As reported earlier, we also found a significant association of asthma with non-availability of separate cooking space and smoking status of father, both of which cause indoor air pollution along with exposure to PAH.[16–18] Several studies have also documented increased prevalence of asthma by indoor air pollution using unprocessed biomass fuels.[3,18–20] Use of unprocessed biomass fuels is a major determinant of indoor air pollution. However, we have not found an association between the uses of biomass fuels with asthma, possibly because of small sample size due to different types of biomass fuels used here. However, ambient air pollution through biomass fuel use is aggravated if there is inadequate ventilation, as found in households that do not have a separate cooking space, which has been found to be associated with asthma in our study. Likewise, we have not found an association of asthma with urban residence, as reported earlier by others.[21]

Because there are no standard guidelines or available data that determine the minimal risk level for a particular PAH for any duration of exposure, any amount of PAH detected in the blood sample was considered as positive by us. In the present study, the mean blood levels of phenanthrene were found to be significantly higher in cases of asthma. Studies have shown that all the PAH detected in our study are found in diesel exhaust particles (DEP), environmental tobacco smoke, and unprocessed biomass fuels.[22] According to the Agency for Toxic Substances and Disease Registry (ATSDR) 1994,[22] the most common PAH in DEP are phenanthrene, fluorenes, fluoranthene, and pyrene, including benzo (a) pyrene, with a major contribution from phenanthrene. We found only a significant association of phenanthrene alone with bronchial asthma and oxidative stress. Recent studies of PAH concentrations in ambient aerosol in Indian cities show that total PAH concentration is 10–50 times higher than that reported internationally.[3,19] Hence, urgent steps are needed to reduce this exposure here.

We have also assessed the blood levels of antioxidant enzymes CAT and SOD in cases and controls. It is quite well known that that loss of CAT activity amplifies oxidative stress and contributes to chronic inflammation.[23–25] Because we recruited cases in the initial phase of acute attack of asthma, we have not observed any difference in the level of CAT between cases and controls. Likewise, SOD in the cytosol is crucial for protecting airways against oxidative stress.[26] SOD converts superoxide anion to hydrogen peroxide.[26] Thereafter, GSH peroxide removes hydrogen peroxide and organic hydroperoxides in a reaction that consumes tripeptide GSH, resulting in a reduction in its levels. We have observed a reduced level of GSH among cases, which can be taken as an evidence of oxidative stress. However, blood levels of SOD were similar in both the groups because during the initial phase of oxidative stress only cytosol levels of SOD are reduced[27] and, with increasing disease duration, this change may also be documented in the blood levels.

This was a matched case control study where simultaneous estimation of PAH and evidence of oxidative stress was performed among cases of bronchial asthma and controls. Therefore, exposure to PAH can be associated with oxidative stress as well as with asthma. We could neither establish dose response of PAH to either the severity of bronchial asthma or oxidative stress. To retain power of 80%, we kept a case to control ratio of 2:1 as it was difficult to obtain consent for venepuncture from healthy controls. Because there are hardly any studies from northern India to show the proportion of population positive for different PAH in blood samples, we only aimed to detect those that were found in at least 50% cases of bronchial asthma and 15% of controls. Thus, our sample size was inadequate to detect an association between PAH and asthma as well as oxidative stress for those that are present in more than 15% of the controls. However, our results can be generalized to other populations.

Because we have documented an elevated blood level of phenanthrene among cases of bronchial asthma along with evidence of oxidative stress, measures to reduce exposure to PAH may possibly lead to reduced incidence and severity of bronchial asthma. However, further studies are needed, with a large sample size, to confirm our findings.

## References

[1] Beasley R (1998). Worldwide variations in prevalence of symptoms of asthma, allergic rhinoconjunctivitis and atopic eczema: ISAAC. The Lancet.

[2] Burney PG, Chinn S, Rana RJ (1990). Has the prevalence of asthma increased in children? Evidence from the national study of health and growth 1973-86. BMJ.

[3] Awasthi S, Kalra E, Roy S, Awasthi S (2004). Prevalence and Risk Factors of Asthma and Wheeze in School-going Children in Lucknow, North India. Indian Pediatr.

[4] Awasthi S, Agarwal S (2003). Determinants of Childhood Mortality and Morbidity in Urban Slums in India. Indian Pediatr.

[5] Gilmour PS, Donaldson K, MacNee W (2002). Overview of the antioxidant pathways in relation to effects of air pollution. Eur Respir Mon.

[6] Pandya RJ, Solomon G, Kinner A, Balmes JR (2002). Diesel exhaust and asthma: Hypotheses and molecular mechanisms of action. Environ Health Perspect.

[7] Leem JH, Kim JH, Hongy, Lee KH, Kang D, Kwon HJ (2005). Asthma attack associated with oxidative stress by exposure to ETS and PAH. J Asthma.

[8] Sachdev HP, Mahajan SC, Garg A (2001). Improving antibiotic and bronchodilator prescription in children presenting with difficult breathing: Experience from an urban hospital in India. Indian Pediatr.

[9] van Schooten FJ, Moonen EJ, van der Wal L, Levels P, Kleinjans JC (1997). Determination of Polycyclic Aromatic Hydrocarbons (PAH) and Their Metabolites in Blood, Feces and Urine of Rats Orally Exposed to PAH Contaminated Soils. Arch Environ Contam Toxicol.

[10] Barranco A, Alonso-Salces RM, Bakkali A, Berrueta LA, Gallo B, Vicente F (2003). Solid-phase clean-up in the liquid chromatographic determination of polycyclic aromatic hydrocarbons in edible oils. J Chromatogr A.

[11] Poon KF, Lam PK, Lam MH (1999). Determination of polynuclear aromatic hydrocarbons in human blood serum by proteolytic digestion-direct immersion SPME. Anal Chim Acta.

[12] Stocks J, Dormandy TL (1971). The autoxidation of human red cell lipids induced by hydrogen peroxide. Br J Haematol.

[13] Jollow DJ, Mitchell JR, Zampaglione N, Gillette JR (1974). Bromobenzene-induced liver necrosis: Protective role of glutathione and evidence for 3, 4-bromobenzene oxide as the hepatotoxic metabolite. Pharmacology.

[14] Sinha AK (1972). Colorimetric assay of catalase. Anal Biochem.

[15] Misra HP, Fridovich I (1972). The role of superoxide anion in the auto-oxidation of epinephrine and a simple assay for superoxide dismutase. J Biol Chem.

[16] Smith KR, Samet JM, Romieu I, Bruce N (2000). Indoor air pollution in developing countries and acute lower respiratory infections in children. Thorax.

[17] Awasthi S, Glick HA, Fletcher RH, Ahmed N (1996). Ambient air pollution and respiratory symptoms complex in preschool children. Indian J Med Res.

[18] Rashidul MH, Kazi SB, Rehman F, Kabir AL, Asif MM, Haque ME (2005). Prevalence of Asthma in highly polluted Dhaka city and low Polluted Coastal area of Bangladesh. Indian J Allergy Asthma Immunol.

[19] Raiyani CV, Jani JP, Desai NM, Shah SH, Shah PG, Kashyap SK (1993). Assessment of indoor exposure to Polycyclic Aromatic Hydrocarbons for urban poor using various types of cooking fuels. Bull Environ Contam Toxicol.

[20] Kulkarni P, Venkataraman C (2000). Atmospheric polycyclic aromatic hydrocarbons in Mumbai, India. Atmospheric Environment.

[21] Davies RJ (1998). Why is allergy increasing? Environmental factors. Clin Exp Allergy.

[22] ASTDR 1944 (1994). Material submitted in support of comments on toxicological profile for PAHs, regulations and advisories. Agency for Toxic Substances and Disease Registry.

[23] Comhair SA, Ricci KS, Arroliga M, Lara AR, Dweik RA, Song W (2005). Correlation of Systemic Superoxide Dismutase Deficiency to Airflow Obstruction in Asthma. Am J Respir Crit Care Med.

[24] Ghosh S, Janocha AJ, Aronica MA, Swaidani S, Comhair SA, Xu W (2006). Nitrotyrosine proteome survey in asthma identifies oxidative mechanism of catalase inactivation. J Immunol.

[25] Wood LG, Gibson PG, Garg ML (2003). Biomarkers of lipid peroxidation, airway inflammation and asthma. Eur Respir J.

[26] Kinnula VL, Crapo JD (2003). Superoxide dismutases in the lung and human lung diseases. Am J Respir Crit Care Med.

[27] Comhair SA, XuW, Ghosh S, Thunnissen FB, Almasan A, Calhoun WJ (2005). Superoxide dismutase inactivation in pathophysiology of asthmatic airway remodeling and reactivity. Am J Pathol.

